# Supplementary material for: Structural distance to the tRNA-synthetase binding interface predicts pathogenicity but is captured by AlphaMissense and EVE
Source: PLoS One. 2026 Nov 11;21(11):e0351982. doi: 10.1371/journal.pone.0351982 (PMC13754165; doi:10.1371/journal.pone.0351982)
Supplement: Supplementary file 1 [file pone.0351982.s001.pdf]

S1 Table. Comparison of data sources. "Unique Proteins" shows how many synthetases are included. "Total Mutations" for UniProt includes variants downloaded from UniProt that were not included in the final analysis due to either a lack of pathogenicity annotation or conflicting annotations; see S2 Table for more detail. "Average Pathogenicity Score" is shown for EVE and Alpha Missense (AM), but not for UniProt or Distance because they do not have quantitative values assigned for pathogenicity of mutations.

Data	EVE	AM	UniProt	Distance
Unique Proteins	14	19	19	13
Total Mutations	203460	283157	16941	8440
Average Pathogenicity Score	0.541	0.599		
Median Pathogenicity Score	0.552	0.661		
