# Supplementary material for: Structural distance to the tRNA-synthetase binding interface predicts pathogenicity but is captured by AlphaMissense and EVE
Source: PLoS One. 2026 Nov 11;21(11):e0351982. doi: 10.1371/journal.pone.0351982 (PMC13754165; doi:10.1371/journal.pone.0351982)
Supplement: Supplementary file 2 [file pone.0351982.s002.pdf]

S2 Table. Mutation data summary. Individual proteins and corresponding variant counts for which structural distance information was available are included. The number of mutations with an Alpha Missense (AM) and EVE score are listed, but EVE lacks data for IARS2 and NARS. UniProt annotations (pathogenic and benign) are also included. "With UniProt annotation" includes any variant with an annotation on UniProt under "clinical significance." Some annotations contained conflicting interpretations of pathogenicity, so "Pathogenic (UniProt)" and "Benign (UniProt)" show the variants with consistent annotations on UniProt; these were the only variants included in the "true" pathogenicity classifications. See methods for how annotations were classified as pathogenic or benign.

Protein name	UniProt ID	Total mutations	With AM score	With EVE score	With UniProt annotation	Pathogenic (UniProt)	Benign (UniProt)
CARS	P49589	730	730	700	9	4	1
DARS	P14868	529	529	424	24	7	0
GARS	P41250	1045	1045	828	245	6	8
HARS	P12081	655	655	519	161	2	6
KARS	Q15046	800	800	560	32	4	2
LARS	Q9P2J5	1124	1124	973	21	3	4
NARS	O43776	577	577	0	19	7	0
QARS	P47897	1194	1194	909	276	5	4
RARS	P54136	721	721	567	27	5	3
TARS	P26639	740	740	640	9	2	4
VARS	P26640	1224	1224	958	54	12	2
WARS	P23381	408	408	311	7	2	1
YARS	P54577	675	675	660	151	4	7
All Proteins		10422	10422	8049	1035	63	42
