# Supplementary material for: Structural distance to the tRNA-synthetase binding interface predicts pathogenicity but is captured by AlphaMissense and EVE
Source: PLoS One. 2026 Nov 11;21(11):e0351982. doi: 10.1371/journal.pone.0351982 (PMC13754165; doi:10.1371/journal.pone.0351982)
Supplement: Supplementary file 3 [file pone.0351982.s003.pdf]

S3 Table. Summary of Sequence Alignment Quality. Comparing aligned distance data sequences to UniProt data sequences. “Total Rows” indicates the number of variants available in the dataset for each protein (this includes only variants that exist both in UniProt/Alpha Missense and in the pdb sequence used for distance calculations); these can include multiple variants in the same position with different substitutions. “Available AA Pairs” represents the number of amino acids paired for each sequence alignment (this includes only one amino acid per position). “Matching AAs” shows the number of amino acid pairs in each sequence alignment that were identical. “Percent Matching” shows the ratio of matching amino acids to total amino acid pairs available.

Protein	Total Rows	Available AA Pairs	Matching AAs	Percent Matching
CARS	730	481	396	82.3
DARS	529	322	253	78.6
GARS	1045	509	440	86.4
HARS	655	322	97	30.1
KARS	800	456	418	91.7
LARS	1124	748	621	83
NARS	577	368	317	86.1
QARS	1194	572	513	89.7
RARS	721	447	374	83.7
TARS	740	468	387	82.7
VARS	1224	776	617	79.5
WARS	408	274	206	75.2
YARS	675	335	284	84.8
