# Supplementary material for: Structural distance to the tRNA-synthetase binding interface predicts pathogenicity but is captured by AlphaMissense and EVE
Source: PLoS One. 2026 Nov 11;21(11):e0351982. doi: 10.1371/journal.pone.0351982 (PMC13754165; doi:10.1371/journal.pone.0351982)
Supplement: Supplementary file 4 [file pone.0351982.s006.pdf]

Reviewer #1:
We would like to thank Reviewer #1 for their thoughtful review. In particular, the suggestion to expand our dataset was the right thing to do, and also made a big difference in our results. Please see below for responses to individual comments.

Comment 1 -
The feature set is surprisingly narrow. While the authors focus solely on Euclidean distance to tRNA nucleotides, protPheMut (https://pubmed.ncbi.nlm.nih.gov/40721360/) and related approaches demonstrate that integrating network dynamics features, energy changes, and physicochemical properties substantially improves phenotypic discrimination, especially for ambiguous variants. The current model may underperform precisely because it ignores these complementary biophysical dimensions.
Response 1 -
We thank the reviewer for this suggestion to consider other features such as those present in protPheMut. However, our paper's scope differs fundamentally from these approaches. Our question is: does structural knowledge of the tRNA-synthetase tRNA complex improve predictions beyond sequence methods. In addition, protPheMut is not designed for complexes, which our question requires. Our narrowly-scoped contribution: structure geometry of the binding interface adds predictive value to ambiguous variants - is informational and complementary to multi-feature approaches.

Comment 2 -
The sample size for the critical "ambiguous interface" subset (n=17) is too small to draw strong conclusions. The simulation extrapolating AUC improvement to n=100, while suggestive, is speculative. The authors should either (a) expand their dataset using additional ARS isoforms or mitochondrial synthetases, or (b) apply external validation with independent databases to strengthen their claims.
Response 2 -
We thank the reviewer for this very valid suggestion. And we agree that adding more data would help validate the findings. In response we considerably expanded our dataset.

We added 7 mitochondrial synthetases and deleted HARS due to its bad sequence alignment quality. We also updated the results using the latest version of Uniprot. As a result our dataset went from 105 to 884 variants (371 pathogenic, 513 benign), with 51 variants that are ambiguous for BOTH Alpha Missense and Eve (instead of the previous 17). After redoing the analysis the sums of the distances to the two key nucleotides, still pathogenic variants are significantly closer to these nucleotides than the benign variants ($p = 1.3e-09$, median difference of 10 Å) (Fig 2B). However, we found that with more data, distance alone was not enough to discriminate between pathogenic and benign. We adjusted the results to reflect this.

Comment 3 -

The static AlphaFold-based distance metric is a coarse proxy for binding disruption. protPheMut's inclusion of elastic network model (ENM)-derived flexibility indices captures conformational dynamics, which may better distinguish pathogenic from benign interface variants. The authors should discuss whether dynamics-based features could supplement or replace Euclidean distance in future iterations.

Response 3 -

Thank you for this great suggestion. We agree that dynamics-based features could supplement or replace Euclidean distance in future iterations, and we have added a comment to the future directions section of our discussion addressing this. However, we would like to note that it is not simple to parameterize a dynamic protein-RNA interaction, and it is outside of the scope of this paper.

Comment 4 -

The clinical utility of the approach remains unclear. The authors should consider presenting decision curve analysis or net reclassification improvement to assess whether adding distance truly benefits patient-level variant interpretation.

Response 4 -

We appreciate this suggestion. After adding significantly more data, based on our AUC analysis of ambiguous-interface variants (Fig 9) and our leave-one-out cross-validation model comparison (Table 1), structural distance does not meaningfully improve discrimination beyond AM+EVE at the individual-variant level: AUC for distance-based classification of ambiguous variants remains near 0.60 regardless of sample size, below the threshold typically considered clinically useful (~0.8), and adding distance to the AM+EVE model does not improve out-of-sample predictive performance (ELPD LOO). Given this, we do not believe decision curve analysis or net reclassification improvement would show meaningful clinical utility for distance as currently measured, and we have revised our conclusions accordingly: rather than proposing distance as a clinically useful feature, we conclude that finer-grained structural or other features (beyond simple Euclidean distance) are needed before structural information can meaningfully inform patient-level variant interpretation (Discussion).

We sincerely thank the reviewer for their careful and constructive comments. The suggestions improved the accuracy of our claims. Thank to their comments, with some work we were able to expand our sample size and that made things more clear. We believe that thanks to this the claims in the manuscript are now stronger.

<u>Reviewer #2:</u>

We would like to thank Reviewer #2 for their feedback. Please see below for responses to individual comments.

Comment 1 - Title evaluation

The title is informative but lengthy and could be shortened. In addition, the term "active site" may be misleading, as the study primarily focuses on the tRNA-binding interface rather than the active site itself. I recommend revising the title to better reflect the focus of the study.

Response 1 -

Thank you for this comment and we agree that the title is long and could be clearer. We have edited the title according to this suggestion.

Comment 2 - Abstract evaluation

The abstract clearly summarises the study. However, the following sentence is too long. Split it into shorter sentences "Incorporating explicit distance information into a Bayesian mixture model did not substantially improve predictive performance over AlphaMissense and EVE alone, suggesting that these models already implicitly capture relevant structural binding context."

Response 2 -

We appreciate this suggestion, and edits to this sentence in the abstract show it is now split into two sentences.

Comment 3 - Introduction

The introduction would be stronger if the author had briefly explained why mutations near the tRNA-binding interface are more likely to be pathogenic. Also, a brief explanation of why AlphaMissense and EVE were selected for this study instead of other pathogenicity prediction tools would suffice.

Response 3 -

We thank the reviewer for this suggestion, and edits addressing these comments have been added to the introduction.

Comment 4 - Results

Pathogenic Variants Cluster Near the ARS-tRNA Interface

- Please include the median difference (or effect size) alongside the p-value to better illustrate the difference between pathogenic and benign variants.

The Bayesian mixture model combined structural distance with existing pathogenic predictors

- Why were AUC, LOO, and MSE chosen to evaluate model performance (biology readers may not be familiar with these statistical measures).

Response 4 -

Thank you for this comment, and we agree that adding effect size would help appreciate more the difference between benign and pathogenic, and more explanation about the performance metrics would help people understand these methods better. We added the median difference and also expanded our explanations in the model performance metrics.

Comment 5 - Discussion

The discussion concludes that structural distance may improve classification scores for ambiguous variants. Can you explain why these variants may benefit from the structural information, while AlphaMissense and EVE perform well for most other variants.

In the future work session, the author suggests incorporating stability and conformational dynamics. How will these improve the pathogenicity prediction?

Response 5 -

We appreciate these suggestions, and these comments are addressed in two parts in the suggested edits in the discussion.

Comment 6 - Methods

The author transformed the data using the Gamma CDF, but they never explained why Gamma was chosen instead of another distribution.

Response 6 -

Thank you for this suggestion, and even though we did include some information on this, it was not very clear. The rationale for the Gamma distribution is presented in the Results section (Figs 3A and 3B), where we show it provided a good fit to the empirical distances to both key nucleotides. We have added a reference to this in the Methods section in order to make this connection clearer.

We would like to thank the reviewer for their constructive comments, the various edits and clarifications significantly improved the manuscript.
