# Supplementary material for: Structural distance to the tRNA-synthetase binding interface predicts pathogenicity but is captured by AlphaMissense and EVE
Source: PLoS One. 2026 Nov 11;21(11):e0351982. doi: 10.1371/journal.pone.0351982 (PMC13754165; doi:10.1371/journal.pone.0351982)
Supplement: Supplementary file 5 [file pone.0351982.s007.pdf]

Reviewer #1: The authors should cite appropriate references in the Discussion and Limitations sections to support their claims.

We thank the reviewer and agree that our claims should be well supported. We went through the entire Discussion and Limitations sections and added citations as follows:

Discussion

Pathogenic Variants Cluster at the ARS-tRNA Interface

- Functionally critical residues are highly conserved and impose conservation gradients on nearby residues (Jack et al, 2016; Ribeiro et al, 2020)
- Mutations near the interface may disrupt tRNA charging, and loss of aminoacylation drives disease (Meyer-Schuman et al, 2017; Oprescu et al, 2017; Kalotay, Klugmann et al Front Neurosci 2023)
- Clinical reports of ARS variants are scarce (Mendes et al, 2026).
- The two models' different score levels reflect their different underlying assumptions (Frazer et al 2021; Cheng et al 2023)

Data and Parameters Used for the New Model

- Bayesian rationale (Gelman et al, 2014)
- Misclassification rates particularly elevated in intrinsically disordered regions where AlphaFold 2 confidence is low (Kong et al, 2025)

Evaluating the New Model

- An ELPD difference within its SE means the models are comparable (Sivula et al, 2025)
- AUC is insensitive to added predictors, and nested-model AUC comparisons need caution (Demler et al. 2012)
- AlphaMissense is trained on AlphaFold2 structures (Cheng et al 2023)
- Eve uses evolutionary constraints that reflect structural conservation (Frazer et al 2021)
- Selective pressure on functionally important sites is reflected in increased conservation in multiple sequence alignments (Jack et al, 2016; Giege et al 2023; Giege et al 1998)
- Proximity being implicitly captured by AI-models (Jack et al, 2016; Frazer et al 2021)

Ambiguous Interface Variants Reveal an Area for Improvement

- AlphaMissense and EVE rely mostly on evolutionary conservation – (Frazer et al, 2021 and Cheng et al 2023)
- Adding different discriminative features like protein stability – (Rodrigues et al, 2021)

Limitations and Future Directions

- Structural alignments can be a source of error – (Sadowski et al, 2012)
- Evolutionary divergence degrades alignment quality – (Kalaimathy et al, 2011)
- The reliance on static structures also fails to capture how mutations influence structural dynamics and protein stability – (Terwilliger et al, 2024, Ose et al, 2022).
- Molecular dynamics simulations could be explored as a complementary tool to capture conformational changes that a single structural snapshot cannot reflect – (Childers et al, 2018; Thompson, Simonson, et al. JBC 2006; Thompson. Plateau, Simonson Chembiochem 2006)
- Protein stability metrics could help to identify variants that disrupt folding – (Redler et al, 2016)
- Variants in understudied genes or underrepresented populations obscure the true distribution (Popejoy et al, 2016)

In addition, we made a few small language modifications to make the statements more accurate.

Reviewer #2: The authors have addressed all my comments. Rather than forcing a narrative, they expanded their dataset substantially and transparently updated their conclusions when the larger dataset revealed the limitations of simple Euclidean distance for ambiguous variants. The revisions enhance the quality and clarity of the paper.

We thank the reviewer for the kind assessment. We expanded the dataset and were transparent about how that affected the results. We agree that these revisions enhanced the paper.
