# Supplementary material for: Structural distance to the tRNA-synthetase binding interface predicts pathogenicity but is captured by AlphaMissense and EVE
Source: PLoS One. 2026 Nov 11;21(11):e0351982. doi: 10.1371/journal.pone.0351982 (PMC13754165; doi:10.1371/journal.pone.0351982)
Supplement: Supplementary file 6 [file pone.0351982.s004.docx]

**Structural distance at the tRNA synthetase active site interface predicts pathogenicity but is captured by AlphaMissense and EVE except among score-ambiguous variants**

## Title evaluation

The title is informative but lengthy and could be shortened. In addition, the term "active site" may be misleading, as the study primarily focuses on the tRNA-binding interface rather than the active site itself. I recommend revising the title to better reflect the focus of the study.

## Abstract evaluation

The abstract clearly summarises the study. However, the following sentence is too long. Split it into shorter sentences “Incorporating explicit distance information into a Bayesian mixture model did not substantially improve predictive performance over AlphaMissense and EVE alone, suggesting that these models already implicitly capture relevant structural binding context.”

## Introduction

The introduction would be stronger if the author had briefly explained why mutations near the tRNA-binding interface are more likely to be pathogenic.

Also, a brief explanation of why AlphaMissense and EVE were selected for this study instead of other pathogenicity prediction tools would suffice.

## Results

**Pathogenic Variants Cluster Near the ARS-tRNA Interface**

Please include the median difference (or effect size) alongside the p-value to better illustrate the difference between pathogenic and benign variants.

**The Bayesian mixture model combined structural distance with existing pathogenic predictors**

Why were AUC, LOO, and MSE chosen to evaluate model performance (biology readers may not be familiar with these statistical measures).

## Discussion

The discussion concludes that structural distance may improve classification scores for ambiguous variants. Can you explain why these variants may benefit from the structural information, while AlphaMissense and EVE perform well for most other variants.

In the future work session, the author suggests incorporating stability and conformational dynamics. How will these improve the pathogenicity prediction?

## Methods

The author transformed the data using the Gamma CDF, but they never explained why Gamma was chosen instead of another distribution.

## Final recommendation

This is a well-designed and well-written study that tackles an important problem in variant pathogenicity prediction. The methodology is sound, the analyses are thorough, and the conclusions are backed up by the results. The revisions are mostly minor and concern clarifying some points and adding more methodological details. I therefore recommend acceptance after minor revision
