# Supplementary material for: Structural distance to the tRNA-synthetase binding interface predicts pathogenicity but is captured by AlphaMissense and EVE
Source: PLoS One. 2026 Nov 11;21(11):e0351982. doi: 10.1371/journal.pone.0351982 (PMC13754165; doi:10.1371/journal.pone.0351982)
Supplement: Peer review history

# Author comment: Author response to Decision Letter 0

**DOI:** 10.1371/journal.pone.0351982.r001

**Transfer Alert**

This paper was transferred from another journal. As a result, its full editorial history (including decision letters, peer reviews and author responses) may not be present.

2 Jun 2026

# Peer review: Decision Letter 0

**DOI:** 10.1371/journal.pone.0351982.r002

**Authors:** Özlem Tastan Bishop

4 Aug 2026

Dear Dr. Barrantes Reynolds,

Thank you for submitting your manuscript to PLOS ONE. After careful consideration, we feel that it has merit but does not fully meet PLOS ONE’s publication criteria as it currently stands. Therefore, we invite you to submit a revised version of the manuscript that addresses the points raised during the review process.

Please submit your revised manuscript by Oct 03 2026 11:59PM. If you will need more time than this to complete your revisions, please reply to this message or contact the journal office at plosone@plos.org. When you're ready to submit your revision, log on to https://www.editorialmanager.com/pone/ and select the 'Submissions Needing Revision' folder to locate your manuscript file.

- A letter that responds to each point raised by the academic editor and reviewer(s). You should upload this letter as a separate file labeled 'Response to Reviewers'.
- A marked-up copy of your manuscript that highlights changes made to the original version. You should upload this as a separate file labeled 'Revised Manuscript with Track Changes'.
- An unmarked version of your revised paper without tracked changes. You should upload this as a separate file labeled 'Manuscript'.

If you would like to make changes to your financial disclosure, please include your updated statement in your cover letter. Guidelines for resubmitting your figure files are available below the reviewer comments at the end of this letter.

If applicable, we recommend that you deposit your laboratory protocols in protocols.io to enhance the reproducibility of your results. Protocols.io assigns your protocol its own identifier (DOI) so that it can be cited independently in the future. For instructions see: https://journals.plos.org/plosone/s/submission-guidelines#loc-laboratory-protocols. Additionally, PLOS ONE offers an option for publishing peer-reviewed Lab Protocol articles, which describe protocols hosted on protocols.io. Read more information on sharing protocols at https://plos.org/protocols?utm_medium=editorial-email&utm_source=authorletters&utm_campaign=protocols.

As the corresponding author, your ORCID iD is verified in the submission system and will appear in the published article. PLOS supports the use of ORCID, and we encourage all coauthors to register for an ORCID iD and use it as well. Please encourage your coauthors to verify their ORCID iD within the submission system before final acceptance, as unverified ORCID iDs will not appear in the published article. *Only*  the individual author can complete the verification step; PLOS staff *cannot*  verify ORCID iDs on behalf of authors.

We look forward to receiving your revised manuscript.

Kind regards,

Özlem Tastan Bishop

Academic Editor

PLOS One

Journal Requirements:

When submitting your revision, we need you to address these additional requirements.

1. Please ensure that your manuscript meets PLOS ONE's style requirements, including those for file naming. The PLOS ONE style templates can be found at

https://journals.plos.org/plosone/s/file?id=wjVg/PLOSOne_formatting_sample_main_body.pdf and

https://journals.plos.org/plosone/s/file?id=ba62/PLOSOne_formatting_sample_title_authors_affiliations.pdf.

2. If the reviewer comments include a recommendation to cite specific previously published works, please review and evaluate these publications to determine whether they are relevant and should be cited. There is no requirement to cite these works unless the editor has indicated otherwise.

[Note: HTML markup is below. Please do not edit.]

Reviewers' comments:

Reviewer's Responses to Questions

**Comments to the Author**

1. Is the manuscript technically sound, and do the data support the conclusions?

Reviewer #1: Yes

Reviewer #2: Yes

**********

2. Has the statistical analysis been performed appropriately and rigorously?

Reviewer #1: Yes

Reviewer #2: Yes

**********

3. Have the authors made all data underlying the findings in their manuscript fully available?

The PLOS Data policy

Reviewer #1: Yes

Reviewer #2: Yes

**********

4. Is the manuscript presented in an intelligible fashion and written in standard English?

Reviewer #1: Yes

Reviewer #2: Yes

**********

Reviewer #1: This study investigates whether explicit structural distance to the tRNA-binding interface improves pathogenicity prediction in aminoacyl-tRNA synthetases (ARSs), beyond what AlphaMissense and EVE already provide. The work is well-motivated, methodologically sound, and clearly written.

However, from the standpoint of current best practices in machine-learning-based variant effect prediction,his manuscript has notable limitations that should be addressed.

First, the feature set is surprisingly narrow. While the authors focus solely on Euclidean distance to tRNA nucleotides, protPheMut (https://pubmed.ncbi.nlm.nih.gov/40721360/) and related approaches demonstrate that integrating network dynamics features, energy changes, and physicochemical properties substantially improves phenotypic discrimination, especially for ambiguous variants. The current model may underperform precisely because it ignores these complementary biophysical dimensions.

Second, the sample size for the critical “ambiguous interface” subset (n=17) is too small to draw strong conclusions. The simulation extrapolating AUC improvement to n=100, while suggestive, is speculative. The authors should either (a) expand their dataset using additional ARS isoforms or mitochondrial synthetases, or (b) apply external validation with independent databases to strengthen their claims.

Third, the static AlphaFold-based distance metric is a coarse proxy for binding disruption. protPheMut’s inclusion of elastic network model (ENM)-derived flexibility indices captures conformational dynamics, which may better distinguish pathogenic from benign interface variants. The authors should discuss whether dynamics-based features could supplement or replace Euclidean distance in future iterations.

Finally, the clinical utility of the approach remains unclear. The authors should consider presenting decision curve analysis or net reclassification improvement to assess whether adding distance truly benefits patient-level variant interpretation.

In summary, the core hypothesis is interesting and biologically plausible, but the current evidence for distance’s added value is weak. Enriching the feature space and expanding the dataset would considerably strengthen the paper.

Reviewer #2: My review does not exceed the 20 000 character limit, but since I have already placed my suggestions in a word document I have decided to upload this as a seperate attachement.

The authors are encouraged to have a look at the document in order to implement my suggestions.

**********

what does this mean?). If published, this will include your full peer review and any attached files.

If you choose “no”, your identity will remain anonymous but your review may still be made public.

**Do you want your identity to be public for this peer review?**  For information about this choice, including consent withdrawal, please see our Privacy Policy

Reviewer #1: No

Reviewer #2: **Yes:**  Krishna Govender

**********

[NOTE: If reviewer comments were submitted as an attachment file, they will be attached to this email and accessible via the submission site. Please log into your account, locate the manuscript record, and check for the action link "View Attachments". If this link does not appear, there are no attachment files.]

To ensure your figures meet our technical requirements, please review our figure guidelines: https://journals.plos.org/plosone/s/figures

You may also use PLOS’s free figure tool, NAAS, to help you prepare publication quality figures: https://journals.plos.org/plosone/s/figures#loc-tools-for-figure-preparation.

NAAS will assess whether your figures meet our technical requirements by comparing each figure against our figure specifications.

Attachment Submitted filename: comments_reviewer.docx

# Author comment: Author response to Decision Letter 1

**DOI:** 10.1371/journal.pone.0351982.r003

21 Sep 2026

Editor:

We thank the Academic Editor for the opportunity to revise. Of the three points highlighted, we have addressed two directly. On sample size, we expanded the dataset from 105 to 884 clinically annotated variants across 19 tRNA synthetases, adding seven mitochondrial enzymes and refreshing the UniProt annotations to the latest version; as a result the score-ambiguous interface subset tripled, from 17 to 51 variants. On dynamics, we have added a discussion of dynamic-based features to the Future Directions section. On the expanded feature set, we have not incorporated additional biophysical features, and we explain our reasoning in Response 1 to Reviewer 1: our scope is specifically whether distance structural knowledge of the synthetase–tRNA complex adds predictive value beyond state-of-the art AI methods. We have clarified this scope in the manuscript. We note that the larger dataset materially changed our findings — distance alone no longer discriminates, or promises to discriminate, within the ambiguous subset — and we have revised our conclusions accordingly.

Reviewer #1:

We would like to thank Reviewer #1 for their thoughtful review. In particular, the suggestion to expand our dataset was the right thing to do, and also made a big difference in our results. Please see below for responses to individual comments.

Comment 1 -

The feature set is surprisingly narrow. While the authors focus solely on Euclidean distance to tRNA nucleotides, protPheMut (https://pubmed.ncbi.nlm.nih.gov/40721360/) and related approaches demonstrate that integrating network dynamics features, energy changes, and physicochemical properties substantially improves phenotypic discrimination, especially for ambiguous variants. The current model may underperform precisely because it ignores these complementary biophysical dimensions.

Response 1 -

We thank the reviewer for this suggestion to consider other features such as those present in protPheMut. However, our paper's scope differs fundamentally from these approaches. Our question is: does structural knowledge of the tRNA-synthetase tRNA complex improve predictions beyond sequence methods. In addition, protPheMut is not designed for complexes, which our question requires. Our narrowly-scoped contribution: structure geometry of the binding interface adds predictive value to ambiguous variants - is informational and complementary to multi-feature approaches.

Comment 2 -

The sample size for the critical “ambiguous interface” subset (n=17) is too small to draw strong conclusions. The simulation extrapolating AUC improvement to n=100, while suggestive, is speculative. The authors should either (a) expand their dataset using additional ARS isoforms or mitochondrial synthetases, or (b) apply external validation with independent databases to strengthen their claims.

Response 2 -

We thank the reviewer for this very valid suggestion. And we agree that adding more data would help validate the findings. In response we considerably expanded our dataset.

We added 7 mitochondrial synthetases and deleted HARS due to its bad sequence alignment quality. We also updated the results using the latest version of Uniprot. As a result our dataset went from 105 to 884 variants (371 pathogenic, 513 benign), with 51 variants that are ambiguous for BOTH Alpha Missense and Eve (instead of the previous 17). After redoing the analysis the sums of the distances to the two key nucleotides, still pathogenic variants are significantly closer to these nucleotides than the benign variants (p = 1.3e-09, median difference of 10 Å) (Fig 2B). However, we found that with more data, distance alone was not enough to discriminate between pathogenic and benign. We adjusted the results to reflect this.

Comment 3 -

The static AlphaFold-based distance metric is a coarse proxy for binding disruption. protPheMut’s inclusion of elastic network model (ENM)-derived flexibility indices captures conformational dynamics, which may better distinguish pathogenic from benign interface variants. The authors should discuss whether dynamics-based features could supplement or replace Euclidean distance in future iterations.

Response 3 -

Thank you for this great suggestion. We agree that dynamics-based features could supplement or replace Euclidean distance in future iterations, and we have added a comment to the future directions section of our discussion addressing this. However, we would like to note that it is not simple to parameterize a dynamic protein-RNA interaction, and it is outside of the scope of this paper.

Comment 4 -

The clinical utility of the approach remains unclear. The authors should consider presenting decision curve analysis or net reclassification improvement to assess whether adding distance truly benefits patient-level variant interpretation.

Response 4 -

We appreciate this suggestion. After adding significantly more data, based on our AUC analysis of ambiguous-interface variants (Fig 9) and our leave-one-out cross-validation model comparison (Table 1), structural distance does not meaningfully improve discrimination beyond AM+EVE at the individual-variant level: AUC for distance-based classification of ambiguous variants remains near 0.60 regardless of sample size, below the threshold typically considered clinically useful (~0.8), and adding distance to the AM+EVE model does not improve out-of-sample predictive performance (ELPD LOO). Given this, we do not believe decision curve analysis or net reclassification improvement would show meaningful clinical utility for distance as currently measured, and we have revised our conclusions accordingly: rather than proposing distance as a clinically useful feature, we conclude that finer-grained structural or other features (beyond simple Euclidean distance) are needed before structural information can meaningfully inform patient-level variant interpretation (Discussion).

We sincerely thank the reviewer for their careful and constructive comments. The suggestions improved the accuracy of our claims. Thank to their comments, with some work we were able to expand our sample size and that made things more clear. We believe that thanks to this the claims in the manuscript are now stronger.

Reviewer #2:

We would like to thank Reviewer #2 for their feedback. Please see below for responses to individual comments.

Comment 1 - Title evaluation

The title is informative but lengthy and could be shortened. In addition, the term “active site” may be misleading, as the study primarily focuses on the tRNA-binding interface rather than the active site itself. I recommend revising the title to better reflect the focus of the study.

Response 1 -

Thank you for this comment and we agree that the title is long and could be clearer. We have edited the title according to this suggestion.

Comment 2 - Abstract evaluation

The abstract clearly summarises the study. However, the following sentence is too long. Split it into shorter sentences “Incorporating explicit distance information into a Bayesian mixture model did not substantially improve predictive performance over AlphaMissense and EVE alone, suggesting that these models already implicitly capture relevant structural binding context.”

Response 2 -

We appreciate this suggestion, and edits to this sentence in the abstract show it is now split into two sentences.

Comment 3 - Introduction

The introduction would be stronger if the author had briefly explained why mutations near the tRNA-binding interface are more likely to be pathogenic. Also, a brief explanation of why AlphaMissense and EVE were selected for this study instead of other pathogenicity prediction tools would suffice.

Response 3 -

We thank the reviewer for this suggestion, and edits addressing these comments have been added to the introduction.

Comment 4 - Results

Pathogenic Variants Cluster Near the ARS-tRNA Interface

Please include the median difference (or effect size) alongside the p-value to better illustrate the difference between pathogenic and benign variants.

The Bayesian mixture model combined structural distance with existing pathogenic predictors

Why were AUC, LOO, and MSE chosen to evaluate model performance (biology readers may not be familiar with these statistical measures).

Response 4 -

Thank you for this comment, and we agree that adding effect size would help appreciate more the difference between benign and pathogenic, and more explanation about the performance metrics would help people understand these methods better. We added the median difference and also expanded our explanations in the model performance metrics.

Comment 5 - Discussion

The discussion concludes that structural distance may improve classification scores for ambiguous variants. Can you explain why these variants may benefit from the structural information, while AlphaMissense and EVE perform well for most other variants.

In the future work session, the author suggests incorporating stability and conformational dynamics. How will these improve the pathogenicity prediction?

Response 5 -

We appreciate these suggestions, and these comments are addressed in two parts in the suggested edits in the discussion.

Comment 6 - Methods

The author transformed the data using the Gamma CDF, but they never explained why Gamma was chosen instead of another distribution.

Response 6 -

Thank you for this suggestion, and even though we did include some information on this, it was not very clear. The rationale for the Gamma distribution is presented in the Results section (Figs 3A and 3B), where we show it provided a good fit to the empirical distances to both key nucleotides. We have added a reference to this in the Methods section in order to make this connection clearer.

We would like to thank the reviewer for their constructive comments, the various edits and clarifications significantly improved the manuscript.

Attachment Submitted filename: Reviewer Comments PLOS One.pdf

# Peer review: Decision Letter 1

**DOI:** 10.1371/journal.pone.0351982.r004

**Authors:** Özlem Tastan Bishop

14 Oct 2026

Dear Dr. Barrantes Reynolds,

Thank you for submitting your manuscript to PLOS One. After careful consideration, we feel that it has merit but does not fully meet PLOS One’s publication criteria as it currently stands. Therefore, we invite you to submit a revised version of the manuscript that addresses the points raised during the review process.

Please submit your revised manuscript by Nov 28 2026 11:59PM. If you will need more time than this to complete your revisions, please reply to this message or contact the journal office at plosone@plos.org. When you're ready to submit your revision, log on to https://www.editorialmanager.com/pone/ and select the 'Submissions Needing Revision' folder to locate your manuscript file.

- A letter that responds to each point raised by the academic editor and reviewer(s). You should upload this letter as a separate file labeled 'Response to Reviewers'.
- A marked-up copy of your manuscript that highlights changes made to the original version. You should upload this as a separate file labeled 'Revised Manuscript with Track Changes'.
- An unmarked version of your revised paper without tracked changes. You should upload this as a separate file labeled 'Manuscript'.

If you would like to make changes to your financial disclosure, please include your updated statement in your cover letter. Guidelines for resubmitting your figure files are available below the reviewer comments at the end of this letter.

If applicable, we recommend that you deposit your laboratory protocols in protocols.io to enhance the reproducibility of your results. Protocols.io assigns your protocol its own identifier (DOI) so that it can be cited independently in the future. For instructions see: https://journals.plos.org/plosone/s/submission-guidelines#loc-laboratory-protocols. Additionally, PLOS One offers an option for publishing peer-reviewed Lab Protocol articles, which describe protocols hosted on protocols.io. Read more information on sharing protocols at https://plos.org/protocols?utm_medium=editorial-email&utm_source=authorletters&utm_campaign=protocols.

As the corresponding author, your ORCID iD is verified in the submission system and will appear in the published article. PLOS supports the use of ORCID, and we encourage all coauthors to register for an ORCID iD and use it as well. Please encourage your coauthors to verify their ORCID iD within the submission system before final acceptance, as unverified ORCID iDs will not appear in the published article. *Only*  the individual author can complete the verification step; PLOS staff *cannot*  verify ORCID iDs on behalf of authors.

We look forward to receiving your revised manuscript.

Kind regards,

Özlem Tastan Bishop

Academic Editor

PLOS One

Journal Requirements:

If the reviewer comments include a recommendation to cite specific previously published works, please review and evaluate these publications to determine whether they are relevant and should be cited. There is no requirement to cite these works unless the editor has indicated otherwise.

Please review your reference list to ensure that it is complete and correct. If you have cited papers that have been retracted, please include the rationale for doing so in the manuscript text, or remove these references and replace them with relevant current references. Any changes to the reference list should be mentioned in the rebuttal letter that accompanies your revised manuscript. If you need to cite a retracted article, indicate the article’s retracted status in the References list and also include a citation and full reference for the retraction notice.

[Note: HTML markup is below. Please do not edit.]

Reviewers' comments:

Reviewer's Responses to Questions

**Comments to the Author**

Reviewer #1: All comments have been addressed

Reviewer #2: All comments have been addressed

**********

2. Is the manuscript technically sound, and do the data support the conclusions?

Reviewer #1: Yes

Reviewer #2: Yes

**********

3. Has the statistical analysis been performed appropriately and rigorously?

Reviewer #1: Yes

Reviewer #2: Yes

**********

4. Have the authors made all data underlying the findings in their manuscript fully available?

The PLOS Data policy

Reviewer #1: Yes

Reviewer #2: Yes

**********

5. Is the manuscript presented in an intelligible fashion and written in standard English?

Reviewer #1: Yes

Reviewer #2: Yes

**********

Reviewer #1: The authors should cite appropriate references in the Discussion and Limitations sections to support their claims.

Reviewer #2: The authors have addressed all my comments. Rather than forcing a narrative, they expanded their dataset substantially and transparently updated their conclusions when the larger dataset revealed the limitations of simple Euclidean distance for ambiguous variants. The revisions enhance the quality and clarity of the paper.

**********

what does this mean?). If published, this will include your full peer review and any attached files.

If you choose “no”, your identity will remain anonymous but your review may still be made public.

**Do you want your identity to be public for this peer review?**  For information about this choice, including consent withdrawal, please see our Privacy Policy

Reviewer #1: No

Reviewer #2: **Yes:**  Krishna Govender

**********

[NOTE: If reviewer comments were submitted as an attachment file, they will be attached to this email and accessible via the submission site. Please log into your account, locate the manuscript record, and check for the action link "View Attachments". If this link does not appear, there are no attachment files.]

To ensure your figures meet our technical requirements, please review our figure guidelines: https://journals.plos.org/plosone/s/figures

You may also use PLOS’s free figure tool, NAAS, to help you prepare publication quality figures: https://journals.plos.org/plosone/s/figures#loc-tools-for-figure-preparation.

NAAS will assess whether your figures meet our technical requirements by comparing each figure against our figure specifications.

# Author comment: Author response to Decision Letter 2

**DOI:** 10.1371/journal.pone.0351982.r005

25 Oct 2026

Reviewer #1: The authors should cite appropriate references in the Discussion and Limitations sections to support their claims.

We thank the reviewer and agree that our claims should be well supported. We went through the entire Discussion and Limitations sections and added citations as follows:

Discussion

Pathogenic Variants Cluster at the ARS-tRNA Interface

• Functionally critical residues are highly conserved and impose conservation gradients on nearby residues (Jack et al, 2016; Ribeiro et al, 2020)

• Mutations near the interface may disrupt tRNA charging, and loss of aminoacylation drives disease (Meyer-Schuman et al, 2017; Oprescu et al, 2017; Kalotay, Klugmann et al Front Neurosci 2023)

• Clinical reports of ARS variants are scarce (Mendes et al, 2026).

• The two models’ different score levels reflect their different underlying assumptions (Frazer et al 2021; Cheng et al 2023)

Data and Parameters Used for the New Model

• Bayesian rationale (Gelman et al, 2014)

• Misclassification rates particularly elevated in intrinsically disordered regions where AlphaFold 2 confidence is low (Kong et al, 2025)

Evaluating the New Model

• An ELPD difference within its SE means the models are comparable (Sivula et al, 2025)

• AUC is insensitive to added predictors, and nested-model AUC comparisons need caution (Demler et al. 2012)

• AlphaMissense is trained on AlphaFold2 structures (Cheng et al 2023)

• Eve uses evolutionary constraints that reflect structural conservation (Frazer et al 2021)

• Selective pressure on functionally important sites is reflected in increased conservation in multiple sequence alignments (Jack et al, 2016; Giege et al 2023; Giege et al 1998)

• Proximity being implicitly captured by AI-models (Jack et al, 2016; Frazer et al 2021)

Ambiguous Interface Variants Reveal an Area for Improvement

• AlphaMissense and EVE rely mostly on evolutionary conservation – (Frazer et al, 2021 and Cheng et al 2023)

• Adding different discriminative features like protein stability – (Rodrigues et al, 2021)

Limitations and Future Directions

• Structural alignments can be a source of error – (Sadowski et al, 2012)

• Evolutionary divergence degrades alignment quality – (Kalaimathy et al, 2011)

• The reliance on static structures also fails to capture how mutations influence structural dynamics and protein stability – (Terwilliger et al, 2024, Ose et al, 2022).

• Molecular dynamics simulations could be explored as a complementary tool to capture conformational changes that a single structural snapshot cannot reflect – (Childers et al, 2018; Thompson, Simonson, et al. JBC 2006; Thompson. Plateau, Simonson Chembiochem 2006)

• Protein stability metrics could help to identify variants that disrupt folding – (Redler et al, 2016)

• Variants in understudied genes or underrepresented populations obscure the true distribution (Popejoy et al, 2016)

In addition, we made a few small language modifications to make the statements more accurate.

Reviewer #2: The authors have addressed all my comments. Rather than forcing a narrative, they expanded their dataset substantially and transparently updated their conclusions when the larger dataset revealed the limitations of simple Euclidean distance for ambiguous variants. The revisions enhance the quality and clarity of the paper.

We thank the reviewer for the kind assessment. We expanded the dataset and were transparent about how that affected the results. We agree that these revisions enhanced the paper.

Attachment Submitted filename: reviewerComments.pdf

# Editor report: Decision Letter 2

**DOI:** 10.1371/journal.pone.0351982.r006

**Authors:** Özlem Tastan Bishop

26 Oct 2026

Structural distance to the tRNA-synthetase binding interface predicts pathogenicity but is captured by AlphaMissense and EVE

PONE-D-26-27203R2

Dear Dr. Barrantes Reynolds,

We’re pleased to inform you that your manuscript has been judged scientifically suitable for publication and will be formally accepted for publication once it meets all outstanding technical requirements.

Within one week, you’ll receive an e-mail detailing the required amendments. When these have been addressed, you’ll receive a formal acceptance letter and your manuscript will be scheduled for publication.

An invoice will be generated when your article is formally accepted. Please note, if your institution has a publishing partnership with PLOS and your article meets the relevant criteria, all or part of your publication costs will be covered. Please make sure your user information is up-to-date by logging into Editorial Manager at Editorial Manager® and clicking the ‘Update My Information' link at the top of the page. For questions related to billing, please contact billing support.

If your institution or institutions have a press office, please notify them about your upcoming paper to help maximize its impact. If they’ll be preparing press materials, please inform our press team as soon as possible -- no later than 48 hours after receiving the formal acceptance. Your manuscript will remain under strict press embargo until 2 pm Eastern Time on the date of publication. For more information, please contact onepress@plos.org.

Kind regards,

Özlem Tastan Bishop

Academic Editor

PLOS One

Additional Editor Comments (optional):

Reviewers' comments:

# Editor report: Acceptance letter

**DOI:** 10.1371/journal.pone.0351982.r007

**Authors:** Özlem Tastan Bishop

PONE-D-26-27203R2

PLOS One

Dear Dr. Barrantes-Reynolds,

I'm pleased to inform you that your manuscript has been deemed suitable for publication in PLOS One. Congratulations! Your manuscript is now being handed over to our production team.

At this stage, our production department will prepare your paper for publication. This includes ensuring the following:

* All references, tables, and figures are properly cited

* All relevant supporting information is included in the manuscript submission,

* There are no issues that prevent the paper from being properly typeset

You will receive further instructions from the production team, including instructions on how to review your proof when it is ready. Please keep in mind that we are working through a large volume of accepted articles, so please give us a few days to review your paper and let you know the next and final steps.

Lastly, if your institution or institutions have a press office, please let them know about your upcoming paper now to help maximize its impact. If they'll be preparing press materials, please inform our press team within the next 48 hours. Your manuscript will remain under strict press embargo until 2 pm Eastern Time on the date of publication. For more information, please contact onepress@plos.org.

You will receive an invoice from PLOS for your publication fee after your manuscript has reached the completed accept phase. If you receive an email requesting payment before acceptance or for any other service, this may be a phishing scheme. Learn how to identify phishing emails and protect your accounts at https://explore.plos.org/phishing.

If we can help with anything else, please email us at customercare@plos.org.

Thank you for submitting your work to PLOS One and supporting open access.

Kind regards,

PLOS One Editorial Office Staff

on behalf of

Prof Özlem Tastan Bishop

Academic Editor

PLOS One
